# The 9aaTAD Is Exclusive Activation Domain in Gal4

**DOI:** 10.1371/journal.pone.0169261

**Published:** 2017-01-05

**Authors:** Martin Piskacek, Marek Havelka, Martina Rezacova, Andrea Knight

**Affiliations:** 1 Laboratory of Cancer Biology and Genetics, Department of Pathological Physiology, Masaryk University Brno, Czech Republic; 2 Gamma Delta T Cell Laboratory, Department of Pathological Physiology, Faculty of Medicine, Masaryk University Brno, Czech Republic; Università degli Studi di Milano, ITALY

## Abstract

The Gal4 protein is a well-known prototypic acidic activator that has multiple activation domains. We have previously identified a new activation domain called the nine amino acid transactivation domain (9aaTAD) in Gal4 protein. The family of the 9aaTAD activators currently comprises over 40 members including p53, MLL, E2A and other members of the Gal4 family; Oaf1, Pip2, Pdr1 and Pdr3. In this study, we revised function of all reported Gal4 activation domains. Surprisingly, we found that beside of the activation domain 9aaTAD none of the previously reported activation domains had considerable transactivation potential and were not involved in the activation of transcription. Our results demonstrated that the 9aaTAD domain is the only decisive activation domain in the Gal4 protein. We found that the artificial peptides included in the original Gal4 constructs were results of an unintended consequence of cloning that were responsible for the artificial transcriptional activity. Importantly, the activation domain 9aaTAD, which is the exclusive activation domain in Gal4, is also the central part of a conserved sequence recognized by the inhibitory protein Gal80. We propose a revision of the Gal4 regulation, in which the activation domain 9aaTAD is directly linked to both activation function and Gal80 mediated inhibition.

## Introduction

The prototypical activators of transcription, such as Gcn4 and Gal4 transcription factors, have been used in molecular biology to study gene regulation in eukaryotes [1–6], following the discovery of the first activator of transcription in prokaryotes, araC [7]. Earlier, the Gcn4, Gal4 and p53 activation domains have been designated as acidic activators [8–11]. Nevertheless, the next studies have demonstrated also the importance of the hydrophobic amino acids for Gcn4 [12–15] and p53 activators [16–23].

There are four activation domains reported for Gal4 protein. Early Gal4 studies reported one weak N-terminal activation domain AD-I (148–196 aa; 13-fold less efficient than the intact Gal4) and one strong C-terminal activation domain AD-II (763–823 aa; as efficient as the intact Gal4) [24]. Similarly to the Gcn4 acidic activator, authors correlated the presence of multiple acidic amino acids with activation function. Initially, a deletion of the 33 amino acid long C-terminus of the Gal4 protein had only a minor effect on the Gal4 ability to activate transcription (as 85% activity was detected in the construct pMA230, Gal4 region 1–848 aa) [24]. In the next study, the same authors showed that their mutant was fully independent on galactose stimulation [25].

However, the results from Melcher and Johnston studies [26,27] clearly showed that the Gal4 C-terminal 34 aa long end represented another activation domain AD-III, which provided the interaction with coactivators of transcription and also with inhibitory protein Gal80. The Gal4 loss-of-function mutant (1–852 aa, deletion of the Gal4 C-terminal 29 aa long end) called gal4-64 supported these findings [28–30].

In the following Gal4 study from Ptashne, a shorter activation domain AD-III (840–857 aa) was declared as the major activation domain in Gal4 protein [31]. However, the shorter activation domain AD-III did not include the region recognized by inhibitory protein Gal80. Furthermore, the authors reported previously that the Gal4 constructs without the activation domain AD-III were strong activators of transcription (AD-II construct CD15XX, 1–823 aa; AD-II construct CDCD13, 1–844 aa) [24].

We have reported a new activation domain for the Gal4 transcription factor, the nine amino acids transactivation domain, 9aaTAD [32]. The activation domains 9aaTAD are created by a tandem of two hydrophobic clusters that are interspersed by a hydrophilic region. As highlighted previously [32], the hydrophobic clusters of the activation domain 9aaTAD are consistent with a broad evidence of hydrophobic patterns reported for Gcn4, p53 and other transcription factors [12–17,22]. The 9aaTAD pattern was reported and the online prediction could be found on www.piskacek.org ([MDENQSTYG] {KRHCGP} [ILVFWM] {KRHCGP} {CGP} {CGP} [ILVFWM] {CGP} {CGP}, Swissprot syntax, and the twelve specific refinement criteria RC1-12) [32,33]. Occasionally, the 9aaTAD function could be significantly enhanced by the adjacent amino acids (one aa at the C-terminus and up to 4 aa at the N-terminus, which included usually one or more hydrophobic amino acids *e*.*g*. as shown for the Msn2 orthologs [32,34]). Therefore the activation domains 9aaTAD could be up to 14 amino acid long, when the contribution of the adjacent amino acids is discernible.

The position of the Gal4 9aa TAD activation domain did not correspond to any of the previously reported activation domains AD-I, AD-II or AD-III from Ptashne lab. Rather, the position of the activation domain 9aaTAD correlated with the Gal80 binding region and with the region missing in the Gal4 loss-of-function mutant gal4-64 (1–852 aa) [26,27].

In this study, we revised function of all reported Gal4 activation domains. Surprisingly, we found that beside of the activation domain 9aaTAD none of the previously reported activation domains was functional. Our results showed that the 9aaTAD domain is the only decisive activation domain in the Gal4 protein.

## Materials and Methods

### Constructs

The construct pBTM116-HA was generated by an insertion of the HA cassette into the *Eco*RI site of the vector pBTM116 (HA cassette nucleotide sequence: TGG CTG—GAATTA—GCC ACC ATG GCT TAC CCA TAC GAT GTT CCA GAT TAC GCT GTC GAG ATA—GAATTC, which render in amino acids sequence: W L—E L—A T M A Y P Y D V P D Y A V E I—E F). The constructs G1-G45 and H1-H45 were generated by PCR and subcloned into pBTM116 *Eco*RI and *Bam*HI sites. All construct have a spacer of three amino acids inserted into the *Eco*RI site; peptide -NNN- (NNN cassette: GAATTC—AATAATAAT, which render in peptide: EF—NNN). All constructs were sequenced by Eurofins Genomics. Further detailed information about constructs, primer sequences are available on the request.

### Assessment of enzyme activities

The β-galactosidase activity was determined in the yeast strain L40 [35,36]. The strain L40 has integrated the *lacZ* reporter driven by the *lexA* operator. In all hybrid assays, we used 2μ vector pBTM116 for generation of the LexA hybrids. The yeast strain L40, the Saccharomyces cerevisiae Genotype: *MATa ade2 his3 leu2 trp1 LYS*::*lexA-HIS3 URA3*::*lexA-LacZ*, is deposited at ATCC (#MYA-3332). For β-galactosidase assays, overnight cultures propagated in YPD medium (1% yeast extract, 2% bactopeptone, 2% glucose) were diluted to an A_600_ of 0.3 and further cultivated for two hours and collected by centrifugation. The crude extracts were prepared by vortexing with glass beads for 3 minutes. The assay was done with 10 ul crude extract in 1ml of 100 mM phosphate buffer pH7 with 10 mM KCl, 1 mM MgSO4 and 0.2% 2-Mercaptoethanol; reaction was started by 200 ul 0.4% ONPG and stopped by 500 ul 1 M CaCO3. The average value of the β-galactosidase activities from three independent experiments is presented as a percentage of the reference with the standard deviation (means and plusmn; SD; n = 3). We standardized all results to previously reported Gal4 construct HaY including merely the activation domain 9aaTAD with the activity set to 100% [34].

### Western blot analysis

The crude cell extracts were prepared in a buffer containing 200 mM Tris-HCl, pH 8.0, 1 mM EDTA, 10% glycerol (v/v), separated by SDS-PAGE, and blotted to nitrocellulose. The immuno-detection of proteins was carried out using mouse anti-HA antibody (#26183, ThermoFisher Sci) or mouse anti-LexA (#306–719, EMD Millipore Corp). The secondary antibodies used were anti-mouse IgG antibodies conjugated with horseradish peroxidase (#A9044, Sigma Aldrich). The proteins were visualized using Pierce ECL (#32106, ThermoFisher Sci) according to the manufacturer’s instructions.

## Results

### Functional and non-functional activation domains in Gal4 activator

**Fig 1:**
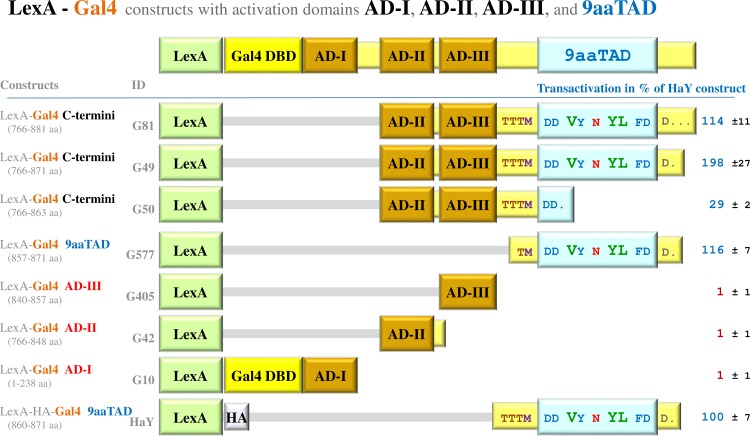
Functional and non-functional activation domains in Gal4 activator. The Gal4—LexA hybrid constructs (BTM116 backbone) were assayed in L40 strain for activation of transcription. To distinguish activity of the N- and C-terminal activation domains in the Gal4 protein, a set of the Gal4 constructs were generated. The average value of the β-galactosidase activities from three independent experiments is presented as a percentage of the reference with standard deviation (means and plusmn; SD; n = 3). We standardized all results to previously reported Gal4 construct HaY including merely the activation domain 9aaTAD with the activity set to 100% [34]. The LexA is *E*.*coli* DNA binding domain and the Gal4 DBD is *S*.*cerevisiae* Gal4 DNA binding domain, both generally used for the generation of fusion hybrids. The regions of Gal4 protein in the constructs are noted and graphically presented. Single dot means end of protein sequence, tree points mean continuing of the sequence, which is no more shown.

**Fig 2:**
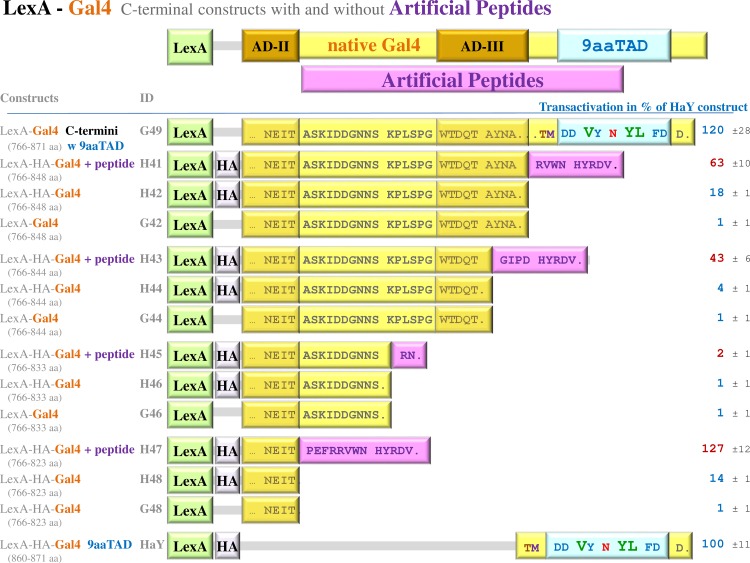
Artificial peptides in the original Gal4 constructs activated transcription. To distinguish natural and artificial activity, a sets of LexA-Gal4 hybrid constructs were generated with and without artificial peptides presented in original constructs, an unintended consequence of cloning [24]. The LexA-Gal4 hybrid constructs assayed in L40 strain for transactivation activity are shown. The average value of the β-galactosidase activities from three independent experiments is presented as a percentage of the reference with standard deviation (means and plusmn; SD; n = 3). We standardized all results to previously reported Gal4 construct HaY including merely the activation domain 9aaTAD with the activity set to 100% [34]. The protein sequences are partially given by amino acid single letter code. Single dot means end of protein sequence, tree points mean continuing of the sequence, which is not more shown. The regions of Gal4 protein in the constructs are noted and graphically presented.

**Fig 3:**
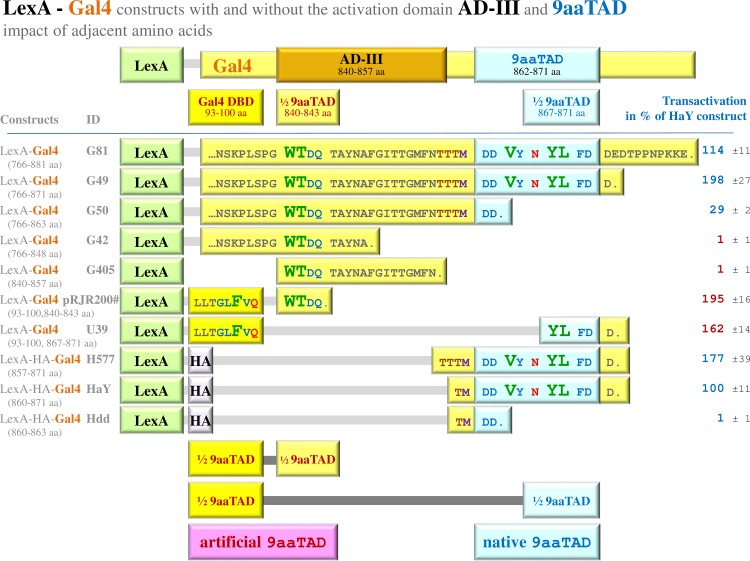
The activation domain AD-III did not activate transcription. The LexA-Gal4 hybrid constructs with activation domains AD-III, 9aaTAD and their artificial variants were assayed in L40 strain for activation of transcription. Gal4 DBD is Gal4 DNA binding domain. The red marks part of the artificial activation domain 9aaTAD (^1^/_2_ 9aaTAD) and the blue marks part of the natural activation domain 9aaTAD (^1^/_2_ 9aaTAD). Both these parts generated strong activators in the fusion with the Gal4 DNA binding domain, Gal4 DBD (83–100 aa), which served as other part of the artificial activation domain 9aaTAD (the second ^1^/_2_ 9aaTAD). The artefact explained previous cloning errors of the reported construct pRJR200 with activation domain AD-III [31]. The impact of proximal amino acids on the function of the activation domain 9aaTAD was tested. The related constructs with and without activation domains AD-III and 9aaTAD are shown again to overview their activities (G81, G49, G59, G42, G405). The average value of the β-galactosidase activities from three independent experiments is presented as a percentage of the reference with standard deviation (means and plusmn; SD; n = 3). We standardized all results to previously reported Gal4 construct HaY including merely the activation domain 9aaTAD with the activity set to 100% [34]. The regions of Gal4 protein in the constructs are noted and graphically presented. The artificial activation domain AD-II is not more shown in this figure. The protein sequences are partially given by amino acid single letter code. Single dot means end of protein sequence, tree points mean continuing of the sequence, which is not more shown.

To we revised function of all reported Gal4 activation domains, we had generated a set of the Gal4 constructs (**Figs 1, 2 and 3)**. The activities of all Gal4 constructs in this study were tested in LexA hybrid assay (the LexA is *E*.*coli* DNA binding domain and the Gal4 DBD is *S*.*cerevisiae* Gal4 DNA binding domain, both generally used for the generation of fusion hybrids; 2μ vector pBTM116 was used for all constructs; strain L40 with integrated lacZ reporter driven by lexA operator was used to assay the activation of transcription). The expression of the LexA-Gal4 protein hybrids were proved by western blotting (**S1 Fig**).

We generated LexA hybrids with all four activation domains of the Gal4 protein, which were reported previously [24,31,32]; the construct G10 (1–238 aa) with the activation domain AD-I (148–238 aa), the construct G42 (766–848 aa) with the activation domains AD-II (766–848 aa), the construct G405 with the activation domain AD-III (840–857 aa), and the construct G577 with the activation domain 9aaTAD (857–871 aa). For the most active C-terminal Gal4 region 766–881 (including the activation domain AD-II, the acid activation domain AD-III and the activation domain 9aaTAD) we generated LexA hybrid series of deletions (766–881, 766–871 and 766–863 aa; constructs G81, G49 and G50).

The constructs G577, G81, and G49, which all included the activation domain 9aaTAD, have the capacity to activate transcription (**Fig 1**). Surprisingly, the constructs G10, G42, G405 and G50, with activation domains AD-I, AD-II or AD-III, had no substantial activity.

### Artificial peptides in the original Gal4 constructs activated transcription

From the data above, it was not clear why the Gal4 activation domains AD-I, AD-II and AD-III were inactive in our constructs. By the inspection of the previously reported data, we identified artificial peptides in the original constructs as a result of an unintended consequence of cloning [24]. Therefore we have generated a set of the Gal4 constructs with and without the artificial peptides (H41, H43, H45, H47 versus H42, H44, H46, and H48). In addition, we generated G42, G44, G46, and G48 constructs without the HA tags (the standard version of LexA hybrid without any modification of the original BTM116 vector). To avoid any cloning artefacts, we have inserted a peptide spacer of three Asparagines (NNN) between the LexA DNA binding domain and the Gal4 fragments.

We have observed that the three constructs with the artificial peptides including the H41 (Gal4 sequence 766–848 aa with RVWN HYRDV peptide), H43 (Gal4 sequence 766–844 aa with GIPD HYRDV peptide) and H47 (Gal4 sequence 766–823 with PEFR RVWN HYRDV peptide) strongly activated transcription (**Fig 2**). The activities of the corresponding constructs without the artificial peptides (constructs H42, H44 and H48) were dramatically reduced or completely diminished. This observation was even more pronounced in the constructs without the HA-tags (constructs G42, G44, G46, and G48). The weak activities were observed in two out of four constructs with the HA-tags (for the constructs H42, H48 but not for H44 and H46 constructs). All constructs without the HA-tags had none substantial activity, therefore the weak activity of the construct H42 (and even less in H48 construct) was considered as an elevated basal level, which extended to 15% of the Gal4 C-terminal construct G49 and 10% of the 9aaTAD construct H577. The LexA-HA construct Hdd with the HA-tag was inactive and therefore the HA-tag could not activate transcription (**Fig 3**).

The artificial peptides in H41, H43 and H47 did not fit the 9aaTAD pattern and therefore are not activation domains of the 9aaTAD family.

### The activation domain AD-III did not activate transcription

In order to fully justify the 9aaTAD domain function, we have generated the Gal4 constructs G49 (776–871 aa) and G50 (776–863 aa) that ended before and after the activation domain 9aaTAD. Furthermore, we have generated two different constructs including merely the activation domain 9aaTAD with two different fusion regions (boundaries) to the LexA DNA binding domain—constructs H577 (857–871 aa) and HaY (860–871 aa) (**Fig 3**). To avoid artefacts, we have inserted the peptide linker of three Asparagines (NNN) between LexA and Gal4 in all our constructs (LexA—HA tag—NNN—Gal4 region).

We have previously reported that fusion of the Gal4 DNA binding domain could occasionally generate strong artificial activation domains [34]. The Gal4 region (92–100 aa) might serve as a half of the artificial activation domain 9aaTAD. We generated construct U39 with a half of the artificial activation domain 9aaTAD originated from the DNA binding domain of the Gal4 (Gal4 region 92–100, peptide LLTGLFVQD) together with four amino acids of the activation domain AD-III, which represented the second half of the artificial activation domain 9aaTAD (Gal4 region 840–843, peptide WTDQ).

As shown in **Fig 3**, the 9aaTAD constructs H577 (857–871 aa) and HaY (860–871 aa) strongly activated transcription comparably with the most active Gal4 C-terminal segments; the constructs G49 (776–871 aa) and G81 (776–881 aa). The activity of the activation domain 9aaTAD is over hundred fold stronger than previously reported the activation domain AD-III as shown for the construct G405 (840–857 aa). The artificial activation domain (construct U39) activated transcription about two hundred fold stronger than the activation domain AD-III (construct G405) and demonstrated a cloning error in the original AD-III construct pRJR200 [31].

## Discussion

Historically, the Gal4 together with Gcn4 and p53 transcription factors represented the acidic activators [1,2,24,25,31,34,37–42]. However, the broad evidence of the essential hydrophobic clusters was reported for Gcn4, p53 and other activators [12–17,22]. Recently, we have reported that both acidic activation domains of the p53 protein are in fact the activation domains 9aaTAD. Moreover, we did not find any conservation of acidic residues in the p53 activation domains 9aaTAD [34].

In this study, we found that none of the previously reported Gal4 activation domains AD-I, AD-II or AD-III was able to activate transcription. Therefore, the domain 9aaTAD is the exclusive activation domain in the Gal4 protein, which has the decisive competence to activate transcription. Thus the term acidic activation domain reported for Gal4 protein [2,25,31] and reported data disproving activation domain 9aaTAD in Gal4 protein [37,43] need urgent revision.

We demonstrated that artificial peptides included in the original Gal4 constructs [24] and fusions of the Gal4 DNA binding domain with random peptides (without proper peptide linker)[31] were responsible for the artificial transcriptional activities in numerous reported Gal4 constructs.

The original Gal4 construct pRJR200 [31] should demonstrate the presence of a strong activation domain AD-III in the Gal4 C-terminal region. In this construct, the Gal4 DNA binding domain (1–100 aa) was fused to the Gal4 region of 18 amino acids (840–857 aa) [31]. Nevertheless, we showed in this study that the same Gal4 region of 18 amino acids was unable to activate transcription (AD-III construct G405). We suspected that an artificial activation domain has been generated in the original AD-III construct pRJR200 by fusion of the Gal4 DNA binding domain with otherwise inactive the Gal4 region AD-III (840–857 aa).

We have previously reported that the fusion of the Gal4 DNA binding domain with random peptides could occasionally generate strong artificial activation domains [34]. The Gal4 region (92–100 aa) serves as a half of the activation domain 9aaTAD. The artefact could be observed in the fusion of either the Gal4 DNA binding domain (1–100 aa) or the small portion (Gal4 region 92–100, peptide LLTGLFVQD-, where hyphen marks fusion site) with the second half of the native Gal4 activation domain 9aaTAD (Gal4 region 867–871, peptide -YLFDD) or with the part of the Gal4 activation domain AD-III (Gal4 region 840–843, peptide -WTDQ).

The latter fusion (construct pRJR200#), the Gal4 DNA binding domain and four amino acids of the Gal4 activation domain AD-III were fused together without any suitable peptide spacer (Gal4 region 92–100 and Gal4 region 840–843, peptide LLTGLFVQD—WTDQ). This fusion was a powerful activator of transcription, which was two-fold stronger than the Gal4 activation domain 9aaTAD (construct HaY). The construct pRJR200# demonstrated presence of a strong artificial activation domain 9aaTAD in the original pRJR200 construct [31].

Similar artificial transactivation domain was generated previously by fusion of the Gal4 DNA binding domain (96–100 aa) and P201 activator [44]. The Gal4-P201 hybrid (peptide LFVQD—YLLPTCIP) was strong activator of transcription [44].

**Fig 4:**
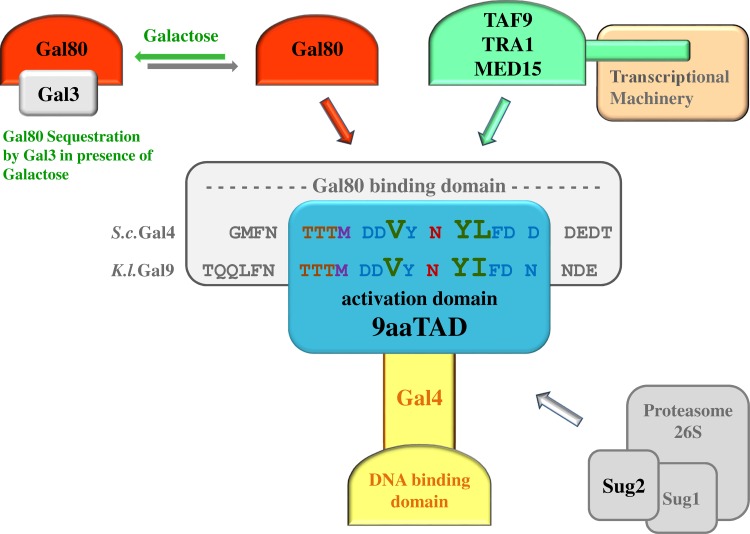
Schema of the Gal4 regulation. Competition of Gal4 inhibitor Gal80 with mediators of transcription for the 9aaTAD domain. The peptides from *S*.*c*.Gal4 and *K*.*l*.Gal9 (Gal4 ortholog) interacting with Gal80 are shown (structural data for peptides interaction at PDB accession code 3E1K and 3BTS). The positions of activation domains 9aaTAD in the Gal80 binding peptides are highlighted (14 amino acid long Gal4 region is needed for maximal activation of transcription, construct H577, including the nine amino acid long 9aaTAD motif and four adjacent amino acids to the N-terminus and one to the C-terminus of the activation domain 9aaTAD). The artificial activation domains AD-I, AD-II and AD-III are not more shown in this figure.

The position of the activation domain 9aaTAD (857–871 aa) correlates with the Gal4 region 854–875, which is necessary and sufficient for the interaction with inhibitory protein Gal80. The 21 aa long peptide derived from the Gal4 region 854–875 tightly binds to the inhibitory protein Gal80 (peptide: GMFNTTTM **DDVYNYLFD** DEDT; the Gal4 activation domain 9aaaTAD was included inside of the peptide: **DDVYNYLFD**; structural data available at PDB accession code 3E1K) [45]. Similarly, related peptide derived from Gal4's ortholog *K*.*l*.Gal9 (peptide: TQQLFNTTTM **DDVYNYIFD** NDE; structural data available at PDB accession code 3BTS) [11] (**Fig 4**). Furthermore, recent report has suggested a functional link between the artificial peptides binding to the Gal80 protein and their ability to activate transcription; "Peptides selected to bind the Gal80 repressor are potent transcriptional activation domains in yeast" [46].

The Gal4 protein is known to interact with MED15 [46], TAF9 [47] and Tra1 [48,49]. Moreover, the Gal4 activator function was linked with MED15 [50–53], Tra1 [54] and SAGA/MED15 complex [55,56]. Importantly, the direct molecular and functional interaction has been reported between the Gal4 activation domain and the Sug2 regulatory protein (subunit of the 26 S proteasome) [57,58]. As a part of the Gal4 regulation, the inhibitory protein Gal80 [28,29,59–61] could be depleted by Gal3 protein in galactose dependent manner [5,62,63]. It has been also shown that the inhibitory protein Gal80 competes with the transcriptional machinery for the same Gal4 region [64,65].

Collectively, we inferred the Gal4 regulation, which involves the activation domain 9aaTAD, the Gal4 mediators of transcription and the inhibitory protein Gal80. We propose a new model of the Gal4 regulation (**Fig 4**) to the previously report [39].

The activation domains 9aaTAD are universally recognized by the transcriptional machinery in eukaryotes. Currently, the 9aaTAD family comprises over 40 members including Gal4, Oaf1, Pip2, Pdr1, Pdr3, Leu3, Tea1, Pho4, Gln3, Gcn4, Msn2, Msn4, Rtg3, E2A, MLL, p53-TAD-I, p53-TAD-II, HNF4 / NHR-49, FOXO3, NF-kB, NFAT, CEBPA/E, ESX, ELF3, ETV1, KLF2/4, EBNA2, VP16, HSF1, HSF2, HsfA, Gli3, Sox18, PIF, Dreb2a, MTF1, OREB1, WRKY45, NS1, MKL1, TRP32, VP16, EBNA2, KBP220, ECapLL, P201, AH, and B42 transcription factors. We and others have shown that the activation domains 9aaTAD have competence to activate transcription as small peptides [32,33,36,66–80]. The activation domains 9aaTAD are annotated on protein database UniProt (http://www.uniprot.org/uniprot/?query=9aatad&sort=score) and the 9aaTAD prediction service is available online (www.piskacek.org).

## Supporting Information

S1 Fig Protein expression. The protein level produced from the Gal4 constructs in L40 strain were monitored by Westernblotting. The proteins comprise LexA a HA tags with a total size of about 21 kDa.
(TIF)
