# Supplementary figures and images for: The 9aaTAD Is Exclusive Activation Domain in Gal4
Source: PLoS One. 2017 Jan 5;12(1):e0169261. doi: 10.1371/journal.pone.0169261 (PMC5215927; doi:10.1371/journal.pone.0169261)

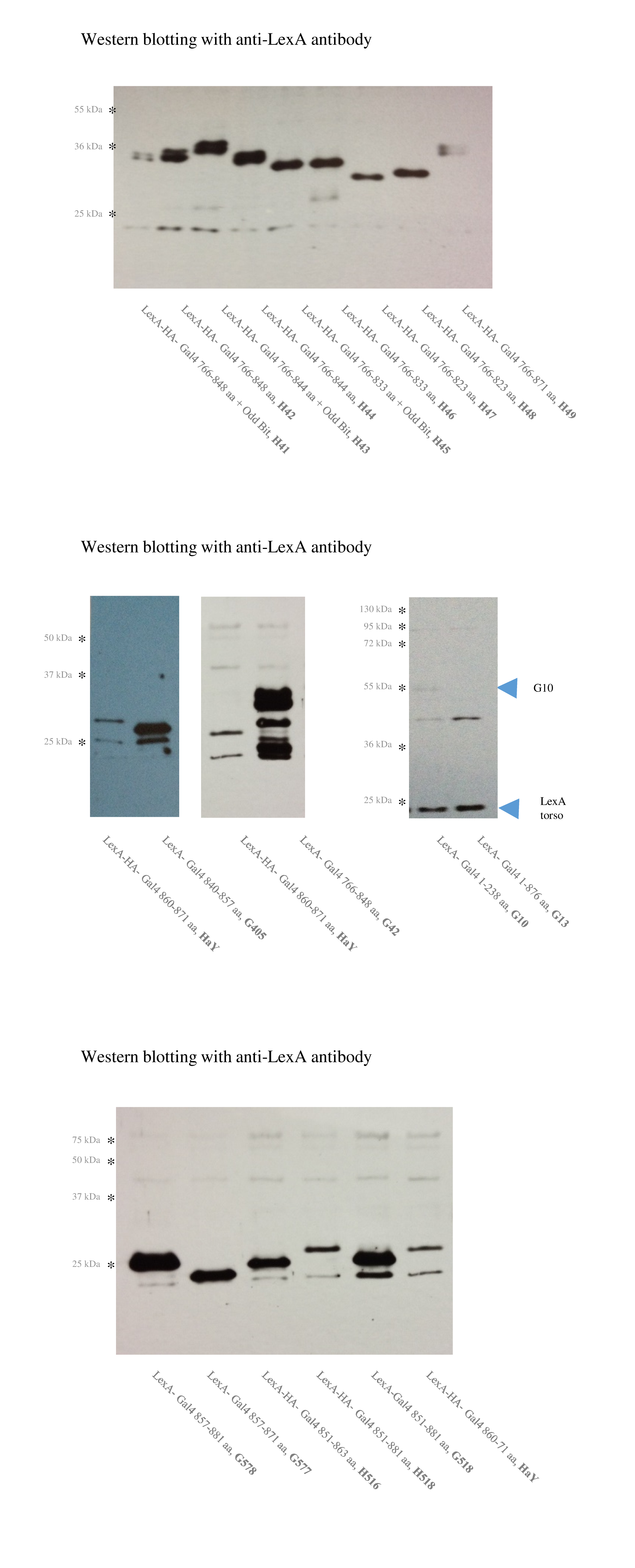

Supplement: S1 Fig — The protein level produced from the Gal4 constructs in L40 strain were monitored by Westernblotting. The proteins comprise LexA a HA tags with a total size of about 21 kDa. (TIF) [file pone.0169261.s001.tif]
